# Coccidia Species and Geographical Distribution in Genus *Sus:* A Scoping Review

**DOI:** 10.3390/microorganisms13010014

**Published:** 2024-12-25

**Authors:** Hongyu Han, Hui Dong, Qiping Zhao, Shunhai Zhu, Bing Huang

**Affiliations:** Key Laboratory of Animal Parasitology of Ministry of Agriculture, Shanghai Veterinary Research Institute, Chinese Academy of Agricultural Sciences, Minhang, Shanghai 200241, Chinadonghui@shvri.ac.cn (H.D.);

**Keywords:** swine coccidiosis, *Eimeria* spp., *Cystoisospora* spp., host, geographical distribution

## Abstract

Swine coccidiosis is a widespread disease caused by species of the apicomplexan parasites *Eimeria* and *Cystoisospora*. Coccidiosis is a common cause of diarrhea in suckling piglets worldwide that directly reduces piglets’ immunity and increases the risk of infection with other enteropathogens, leading to increased clinical infection and mortality and consequent economic losses in the global pig industry. We searched the available literature to date, including English and Chinese articles, using six electronic bibliographic databases, including PubMed, ScienceDirect, Web of Science, CNKI, VIP Chinese Journal Database, and Wanfang Data. A standard approach for conducting scoping reviews was used to identify studies on the species and distribution of genus *Sus* coccidia worldwide. A quality assessment was done for each study reviewed and relevant information reported in the identified studies was collated, categorized, and summarized. A total of 149 publications and references were eligible for the final review. The distribution of 18 species of genus *Sus* coccidia recorded in 63 countries was collated. These included 15 *Eimeria* and 3 *Cystoisospora* species. *C. suis* was found in 48 countries, *E. debliecki* in 45 countries, *E*. *scabra* in 33 countries, *E. polita* in 31 countries, *E. suis* in 28 countries, *E. perminuta* in 26 countries, *E. porci* in 24 countries, *E. neodebliecki* and *E.spinosa* in 21 countries each, *E.guevarai* in 5 countries, *C. almataensis* in 4 countries, *E. betica* in 2 countries, and *E. almataensis*, *E. ibrahimovae*, *E. residualis*, *E. szechuanensis*, *E. yanglingensis*, and *C. sundarbanensis* were each found in only 1 country. Each species was listed according to its scientific name, host name, finding location, and geographical distribution. This review reflects the distribution and infection of genus *Sus* coccidia worldwide and provides more complete basic information to aid our understanding of the species and geographical distribution of coccidia in the genus *Sus*.

## 1. Introduction

Pigs belong to the genus *Sus*, within the even-toed ungulate family Suidae. Pigs include domestic pigs and their ancestor, the common Eurasian wild boar (*Sus scrofa*), along with other species. The genus *Sus* is currently thought to contain eight living species (Table S1) [1]. Domestic pigs are small, adaptable, grow rapidly, are multiparous, and are reared globally for the production of pork and the provision of raw biomedical materials [2]. Pork is the main type of meat consumed globally, with global pork production expected to reach 124.6 million tons in 2022 [3]. The EU is the world’s second-largest producer of pork (after China) and the greatest exporter of pork and pork products, with grossly half of its total meat production coming from pigs [4]. Economic development has led to improvements in the raising environment and health status of pigs in large-scale pig farms during the last two decades. Although many major diseases, such as brucellosis and foot-and-mouth disease, have been eliminated, several milder endemic diseases, such as coccidiosis, may still have significant impacts on production costs [5].

Swine coccidiosis is a widespread disease caused by infection with the apicomplexan parasites *Eimeria* and *Cystoisospora*. Coccidiosis is a common cause of diarrhea in suckling piglets worldwide, associated with the loss of intestinal villi, leading to impaired digestion and food absorption [6]. Infection with *Cystoisospora suis* and *Eimeria* species is common in piglets, with high prevalence rates worldwide [7,8,9,10]. For example, *C. suis* and *Eimeria* spp. were detected in Swedish pigs at 60% and 64% of farms, with the highest prevalences in post-weaning piglets and sows, respectively [7]. In other European countries (Austria, the Czech Republic, Germany, and Spain), overall, 71.4% of farms and 50.1% of litters were positive for *C. suis*, with a positivity rate on some farms up to 100% [8]. A previous report also showed that the prevalence of coccidia infection was about 76% in feral swine (*S. scrofa*) from the Great Smoky Mountains National Park, USA, with coccidia being the second most common type of parasite detected [11].

*Cystoisospora suis* is recognized as an important pathogen of neonatal pigs, causing diarrhea and dehydration, mainly in animals of 2–4 weeks of old. Piglets infected with *C. suis* had shorter intestinal villi and decreased body weight gains, both during the occurrence of clinical symptoms and also after the weaning period (6–7 weeks of age) [6]. Although *Eimeria* species are thought to be less pathogenic, they may also cause clinical signs such as fever, diarrhea, and weight loss after weaning or during the fattening period, especially infections with *Eimeria suis*, *Eimeria polita*, and *Eimeria spinosa*. On the other hand, *Eimeria* infections can serve as an indicator of the overall hygiene within a farm [12,13].

Despite the low mortality of pigs due to coccidiosis, it can lead to significant economic losses as a result of decreased daily weight gain (up to 20% weight loss), feed conversion, and impaired performances of infected animals in subsequent production phases [14]. Previous reports showed an about 15% decrease in body weight gain during coccidiosis, resulting in a lower weaning weight of 500 g per head, and potentially a loss of about 1 kg per head [6]. In addition, coccidiosis directly reduces piglets’ immunity and increases infection with other enteropathogens, such as rotavirus, transmissible gastroenteritis virus, clostridia, and *Escherichia coli*, leading to increased clinical infection and mortality [15].

Pigs have many advantages, including short generation intervals, a high breeding rate, and fast growth, associated with rapid economic benefits to farmers. Especially in low-income countries, farmers thus prefer to keep pigs because they do not require much physical labor to manage, while also having the advantages of economy of space and easy marketing. For example, pigs are the most popular livestock in some areas of Nepal and have traditional, religious, and economic values. Rearing pigs in rural regions is a common practice for subsistence farming [16]; however, most pigs are kept in unhygienic conditions due to the traditional smallholder system, thus increasing the chances of coccidia infection and leading to significant economic loss to the farmers.

This scoping literature review aimed to document coccidia species and their distribution in the genus *Sus* worldwide. We collected relevant literature and present an alphabetical list of coccidia distributed in the genus *Sus* worldwide, according to coccidia species in the genus *Sus* recorded by Levine (1988) [17] and new coccidia species reported by relevant countries [18,19,20].

## 2. Materials and Methods

### 2.1. Literature Search Strategy

We searched the available literature, including English and Chinese articles, using six electronic bibliographic databases: PubMed, ScienceDirect, Web of Science, CNKI, VIP Chinese Journal Database, and Wanfang Data. We used the following keywords: “pig and coccidiosis”, “pig and *Eimeria*”, “pig and *Isospora*”, “pig and *Cystoisospora*”, “*Sus* and coccidiosis”, “*Sus* and *Eimeria*”, “*Sus* and *Isospora*”, and “*Sus* and *Cystoisospora*” for the PubMed, ScienceDirect, and Web of Science databases. For the Chinese databases, we used the Chinese translation of the keywords “pig and coccidia”. There was no restriction on the publication year.

### 2.2. Study Screening and Selection Criteria

We organized the articles using Endnote software X9. Duplicate articles were removed. Three investigators (HHY, ZSH, and ZQP) independently screened all the titles and abstracts to determine if the articles met the inclusion criteria, and articles satisfying the criteria were saved as potential documents (first screening). Any disagreement was mediated by a fourth author (DH), and failure to reach a consensus was resolved by the senior author (HB).

Papers were excluded if they met the following criteria: not related to swine coccidia, biology, and pathogenicity; experimental reports; medicines; establishment of molecular detection methods; life history; English books; and Chinese articles including translations of foreign literature.

The inclusion criteria were: epidemiological investigation and species identification of pig coccidia, including *Eimeria*, *Isospora*, and *Cystoisospora*; sufficient details (synonym, host, parasite location, and geographical distribution); and accessible full texts or abstracts.

Two authors (HHY and DH) independently extracted information from the eligible studies, including article title, first author, year of publication, country of study, epidemiological investigation, species, surveillance, and prevalence. If the same species were found in the same area in different articles or abstracts, the papers that described more species and were more reliable were used for reference in this review. Any discrepancies in the final identified records were discussed; if no consensus was reached, advice was sought from the senior author (HB).

### 2.3. Description of Coccidia Species

Coccidia species in the genus *Sus* were accepted according to previous reports [17,18,19,20]. *Eimeria* species that were accepted as valid were chosen according to taxonomic summaries, including the color and shape of oocysts, roughness or smoothness of the outer layer of the oocyst wall, morphology of oocysts and sporocysts, oocysts residuum absent or present, stieda body in sporocysts absence or presence, etc. [21]. Each species was listed and recorded according to its scientific name, synonym, host name, and finding location, and geographical distribution was arranged by the alphabet of English names of the country or region.

## 3. Results and Discussion

**Figure 1:**
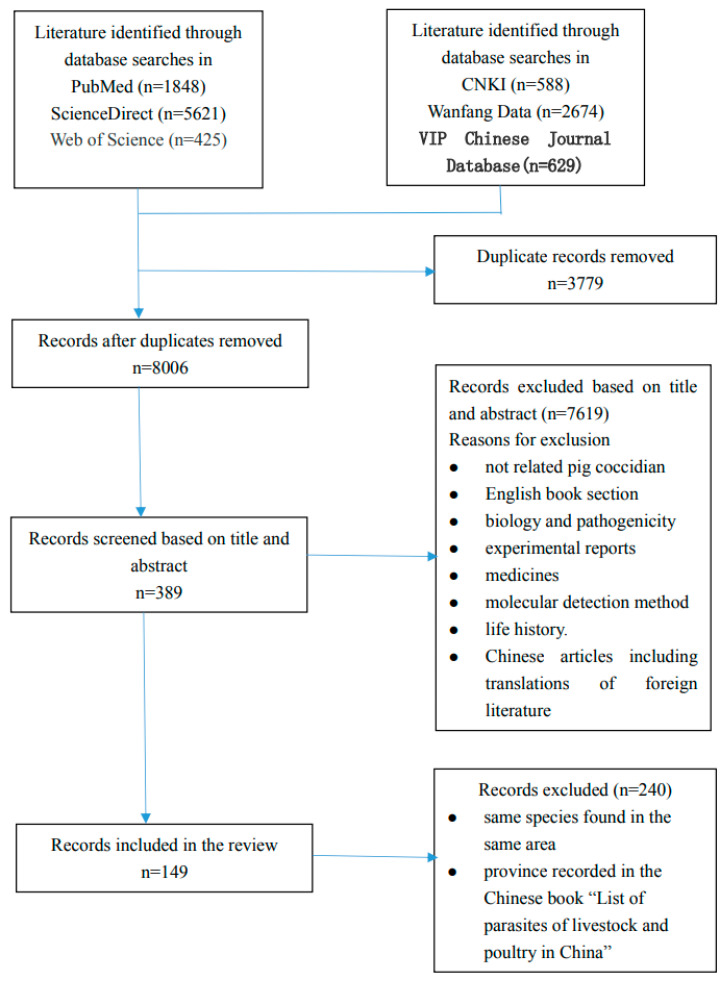
Flow diagram of the literature search and selection process for listing coccidia species in the genus *Sus* around the world by 2024. The number of publications was identified through searching in each database and was obtained after removing duplicates and applying each eligibility criterion.

We obtained 11,785 papers, including articles, abstracts, and book sections, from the six electronic databases. After removing 3779 duplicates, 8006 were screened, and 389 papers including full texts and abstracts were eligible for selection based on the title and abstract relevance. We excluded a further 240 papers because they contained the same species found in the same area in different papers, or because the provinces were recorded in the Chinese book “List of parasites of livestock and poultry in China”. Finally, 149 articles met the inclusion criteria (Figure 1).

### 3.1. Species and Distribution

#### 3.1.1. *Eimeria almataensis* Musaev, 1970

Synonym: *Eimeria debliecki* Douwes, 1921 of Svanbaev (1958) [17].

Host: *Sus scrofa* [22].

Founding location: Oocysts found in feces [17].

Geographic distribution: Kazakhstan (Alma-Ata) [22].

#### 3.1.2. *Eimeria betica* Martinez and Hernandez, 1973

Host: *Sus scrofa* [17].

Founding location: Oocysts found in feces [23].

Geographic distribution: Spain (Cordoba) [24]; Russia (Udmurt) [23].

#### 3.1.3. *Eimeria debliecki* Douwes, 1921

Synonym: *Coccidium suis* Jaeger, 1921; *Eimeria brumpti* Cauchemez, 1921 in part; *Eimeria jalina* Krediet, 1921; *Eimeria scrofae* Galli-Valerio, 1935; *Eimeria polita* Pellérdy, 1949 in part [17].

Host: *Sus scrofa domestica* [25], *Sus scrofa* [26], Sus bucculentus* [27].

Parasitic site: Small intestine [17].

Geographic distribution: Australia (Queensland) [28]; Austria [29]; Azerbaijan (Greater Caucasus) [30]; Belarus [27]; Belgium [26]; Brazil (Maranhão, Paraíba, Rio de Janeiro, Santa Catarina, Uberlândia) [31,32,33,34,35]; Bulgaria [36]; Canada (Quebec) [37]; Chile [38]; China (Anhui, Beijing, Chongqing, Fujian, Gansu, Guangdong, Guangxi, Guizhou, Hebei, Henan, Heilongjiang, Hubei, Hunan, Inner Mongolia, Jiangsu, Jiangxi, Liaoning, Qinghai, Shaanxi, Shandong, Sichuan, Tianjin, Yunnan, Zhejiang) [39,40,41,42,43]; Estonia [44]; France [45]; Germany (Lower Saxony) [46]; Hungary (Mátra) [47]; India (Karnātaka, Meghalaya, Punjab) [48,49,50]; Indonesia (Java) [45]; Italy (Pisa) [51]; Japan (Ibaraki) [52]; Kazakhstan [26]; Kenya (Western Province) [53]; Latvia [54]; Lithuania [55]; Moldova [56]; Nepal (Chitwan) [2]; Netherlands [57]; New Zealand (Wairarapa) [58]; Nigeria (Admawa, Ibadan) [59,60]; Norway (Buskerud) [61]; Papua New Guinea (Port Moresby) [62]; Philippines [63]; Poland (Bialystok, Bochnia, Bydgoskie, Bydgoszcz, Gdańskie, Kieleckie, Łódzkie, Lubelskie, Lublin, Olsztyn, Olsztyńskie, Poznań, Poznańskie, Rzeszów, Rzeszowskie, Warsaw, Wielkopolska, Zielona Góra, Zielonogrskie) [64,65,66,67,68,69]; Romania (Cluj) [13]; Russia (Smolensk, Udmurt) [22,70]; Serbia (Aleksa Šantić, Priština) [71,72]; South Korea (Jeju) [73]; Sri Lanka (Peradeniya) [74]; Switzerland [75]; Tanzania (Morogoro) [76]; Ukraine [77]; United Kingdom (North Yorkshire) [78]; Uruguay [79]; USA (California, Florida, Hawaii, Illinois, Iowa, Kansas, Maryland, Oregon) [25,26,80,81,82,83]; Venezuela [75]; Vietnam (Hanoi) [84]; Zimbabwe (Harare) [85].

* *Sus bucculentus* Heude, 1892–Heude’s pig or Indochinese (or Vietnam) warty pig (possibly extinct since the late 20th–early 21st century; dubious species, may be synonymous with *S. scrofa*) [1].

#### 3.1.4. *Eimeria guevarai* Romero Rodriguez and Lizcano Herrera, 1971

Host: *Sus scrofa* [17].

Founding location: Oocysts found in feces [17].

Geographic distribution: China (Inner Mongolia) [39]; Estonia [44]; Lithuania [55]; Serbia (Priština) [72]; Ukraine [77].

#### 3.1.5. *Eimeria ibrahimovae* Musaev, 1970

Synonym: *Eimeria scabra* Henry, 1931 of Svanbaev (1958); *Eimeria ibragimovae* Musaev, 1970 of Svanbaev (1979) [17].

Host: *Sus scrofa* [17].

Founding location: Oocysts found in feces [17].

Geographic distribution: Kazakhstan (Alma-Ata) [22];

#### 3.1.6. *Eimeria neodebliecki* Vetterling, 1965

Synonym: *Eimeria brumpti* Cauchemez, 1921 in part; *Eimeria debliecki* Douwes, 1921 in part [86].

Host: *Sus scrofa domestica, Sus scrofa scrofa* [26].

Founding location: Oocysts found in feces [26].

Geographic distribution: Australia (Queensland) [87]; Brazil (Paraíba, Rio de Janeiro, Santa Catarina) [32,33,34]; Chile [38]; China (Anhui, Chongqing, Fujian, Guangdong, Henan, Hunan, Jiangsu, Jiangxi, Inner Mongolia, Qinghai, Shaanxi, Shandong, Sichuan, Yunnan, Zhejiang) [39,40,41,88,89]; Estonia [44]; Hungary (Mátra) [47]; India (Punjab, West Bengal) [50,90]; Iran (Mazandaran) [91]; Japan (Ibaraki) [52]; Lithuania [55]; Nepal (Chitwan) [2]; New Zealand (Wairarapa) [58]; Nigeria (Ibadan) [60]; Papua New Guinea (Port Moresby) [62]; Russia [92]; Sri Lanka (Peradeniya) [74]; Tanzania (Morogoro) [76]; Ukraine [77]; United Kingdom (North Yorkshire) [78]; USA (Florida, Georgia, Illinois, Kansas, Oregon, Utah) [26,80,81,83]; Zimbabwe (Harare) [85].

#### 3.1.7. *Eimeria perminuta* Henry, 1931

Synonym: *Eimeria perminuta* var. *mathurai* Mishra, 1967 [86].

Host: *Sus scrofa domestica* [26].

Founding location: Oocysts found in feces [17].

Geographic distribution: Azerbaijan (Greater Caucasus) [30]; Brazil (Maranhão, Paraíba, Santa Catarina, Uberlândia) [31,32,34,35]; China (Anhui, Beijing, Chongqing, Fujian, Guangxi, Guizhou, Hebei, Henan, Hunan, Jiangsu, Jiangxi, Inner Mongolia, Qinghai, Shaanxi, Sichuan, Tianjin, Tibet, Yunnan, Zhejiang) [39,40,43,88,93,94]; Colombia (Caldas) [95]; Germany (Lower Saxony) [46]; Hungary (Mátra) [47]; India (Karnātaka, Meghalaya, Punjab) [48,49,50]; Japan (Ibaraki) [52]; Latvia [54]; Lithuania [55]; Moldova [56]; Nepal (Chitwan) [2]; Nigeria (Admawa, Ibadan) [59,60]; Papua New Guinea (Port Moresby) [62]; Poland (Bialystok, Bochnia, Wielkopolska) [64,65,69]; Romania [25]; Russia (Smolensk) [70]; Serbia (Aleksa Šantić) [71]; South Korea (Jeju) [73]; Tanzania (Morogoro) [76]; Ukraine [77]; United Kingdom (North Yorkshire) [78]; Uruguay [79]; USA (California, Florida, Illinois, Kansas) [26,80,81]; Vietnam (Hanoi) [84]; Zimbabwe (Harare) [85].

#### 3.1.8. *Eimeria polita* Pellérdy, 1949

Synonym: *Eimeria cerdonis* Vetterling, 1965 [17]; *Eimeria debliecki* Douwes, 1921 in part [86].

Host: *Sus scrofa domestica* [96], *Sus scrofa* [97].

Parasitic site: Small intestine [17].

Geographic distribution: Australia (Queensland) [28]; Austria [29]; Azerbaijan (Greater Caucasus) [30] Brazil (Maranhão, Paraíba, Rio de Janeiro, Uberlândia) [31,32,33,35]; Chile [38]; China (Anhui, Beijing, Chongqing, Fujian, Guangxi, Guizhou, Hebei, Henan, Hunan, Jiangsu, Jiangxi, Qinghai, Shaanxi, Sichuan, Tianjin, Tibet, Yunnan, Zhejiang) [39,40,41,42,43,88,93,98]; Colombia (Caldas) [95]; Estonia [44]; Germany (Lower Saxony) [46]; Hungary [75]; India (Meghalaya, Punjab) [49,50]; Japan (Ibaraki) [52]; Kenya (Western Province) [53]; Latvia [54]; Lithuania [55]; Nepal (Chitwan) [2]; New Zealand (Wairarapa) [58]; Nigeria (Admawa, Ibadan) [59,60]; Norway (Buskerud) [61]; Papua New Guinea (Port Moresby) [62]; Poland (Bialystok, Bochnia, Bydgoskie, Bydgoszcz, Gdańskie, Kieleckie, Łódzkie, Lublin, Lubelskie, Olsztyn, Olsztyn, Olsztyńskie, Poznań, Poznańskie, Rzeszów, Rzeszowskie, Warsaw, Zielona Góra, Zielonogrskie) [63,64,65,66,67]; Romania [26]; Russia (Smolensk) [70]; Serbia (Aleksa Šantić) [71]; South Korea (Jeju) [73]; Tanzania (Morogoro) [76]; Ukraine [77]; United Kingdom (North Yorkshire) [78]; Uruguay [79]; USA (Alabama, Florida, Georgia, Illinois, Kansas, Maryland) [26,75,80,81,82]; Zimbabwe (Harare) [85].

#### 3.1.9. *Eimeria porci* Vetterling, 1965

Synonym: *Eimeria debliecki* Douwes, 1921 in part [86].

Host: *Sus scrofa domestica* [26], *Sus scrofa scrofa* [99].

Parasitic site: Jejunum and ileum [17].

Geographic distribution: Australia (Queensland) [87]; Austria [99]; Brazil (Maranhão, Paraíba, Rio de Janeiro, Santa Catarina) [31,32,33,34]; Chile [38]; China (Anhui, Beijing, Chongqing, Fujian, Guangxi, Guizhou, Hebei, Henan, Hunan, Jiangsu, Qinghai, Shaanxi, Sichuan, Tianjin, Tibet, Yunnan) [39,40,41,42,43,88,93,94]; Colombia (Caldas) [95]; Estonia [44]; Germany (Lower Saxony) [46]; India (Meghalaya, Punjab) [49,50]; Iran (Mazandaran) [91]; Japan (Ibaraki) [52]; Kenya (Western Province) [53]; Lithuania [55]; Nepal (Chitwan) [2]; New Zealand (Wairarapa) [58]; Nigeria (Admawa, Ibadan) [59,60]; Papua New Guinea (Port Moresby) [62]; Russia [92]; Sri Lanka (Peradeniya) [74]; Tanzania (Morogoro) [76]; United Kingdom (North Yorkshire) [78]; Uruguay [79]; USA (Florida, Illinois, Kansas, Maryland, Utah) [26,80,81,82,83]; Zimbabwe (Harare) [85].

#### 3.1.10. *Eimeria residualis* Martinez and Hernandez, 1973

Host: *Sus scrofa* [17].

Founding location: Oocysts found in feces [17].

Geographic distribution: Spain (Cordoba) [24].

#### 3.1.11. *Eimeria scabra* Henry, 1931

Synonym: *Eimeria debliecki* Douwes, 1921 in part [86]; *Eimeria romaniae* Donçiu, 1962 [17]; *Eimeria scarba* of Yakimoff and Matikaschwili (1932) *lapsus calami* [17].

Host: *Sus scrofa* [77], *Sus scrofa domestica* [25].

Parasitic site: Small intestine [17], large intestine [25].

Geographic distribution: Australia (Queensland) [28]; Austria [29]; Azerbaijan (Greater Caucasus) [30]; Brazil (Maranhão, Paraíba, Piaui, Rio de Janeiro, Santa Catarina, Uberlândia) [31,32,33,34,35,100]; Bulgaria [36]; Chile [38]; China (Anhui, Beijing, Chongqing, Fujian, Guangdong, Guangxi, Guizhou, Hebei, Henan, Hubei, Hunan, Jiangsu, Jiangxi, Liaoning, Inner Mongolia, Qinghai, Shaanxi, Shandong, Sichuan, Tianjin, Tibet, Yunnan, Zhejiang) [39,40,41,42,43,88,94]; Congo [26]; Czech (České Budĕjovice) [101]; Germany (Lower Saxony) [46]; Hungary (Mátra) [47]; India (Karnātaka, P unjab) [48,50]; Japan (Ibaraki) [52]; Kenya (Western Province) [53]; Latvia [54]; Lithuania [55]; Moldova [56]; Nepal (Chitwan) [2]; New Zealand (Wairarapa) [58]; Nigeria (Admawa, Ibadan) [59,60]; Papua New Guinea (Port Moresby) [62]; Poland (Bochnia, Bydgoskie, Bydgoszcz, Kieleckie, Łódzkie, Lubelskie, Lublin, Olsztyn, Olsztyńskie, Poznań, Poznańskie, Rzeszów, Rzeszowskie, Warsaw, Wielkopolska, Zielona Góra, Zielonogrskie) [65,66,67,68,69]; Romania [26]; Russia (Smolensk) [70]; Serbia (Priština) [72]; South Korea (Jeju) [73]; Sri Lanka (Peradeniya) [69]; Tanzania (Morogoro) [76]; Ukraine [77]; Uruguay [79]; USA (California, Florida, Georgia, Hawaii, Illinois, Kansas, Oregon, Utah) [25,26,80,81,83,102]; Vietnam (Hanoi) [84]; Zimbabwe (Harare) [85].

#### 3.1.12. *Eimeria spinosa* Henry, 1931

Host: *Sus scrofa domestica* [25], *Sus scrofa scrofa* [99].

Parasitic site: Small intestine [26], large intestine [24].

Geographic distribution: Austria [29]; Brazil (Santa Catarina) [34]; Bulgaria [36]; China (Anhui, Beijing, Chongqing, Fujian, Hebei, Henan, Hubei, Hunan, Jiangsu, Tianjin, Tibet, Yunnan) [39,94]; Colombia (Caldas) [95]; Czech (České Budĕjovice) [103]; Germany (Lower Saxony) [45]; India (Meghalaya, Punjab) [49,50]; Japan (Ibaraki) [52]; Latvia [54]; Lithuania [55]; Moldova [56]; Netherlands [57]; Nigeria (Admawa, Ibadan) [59,60]; Papua New Guinea (Port Moresby) [62]; Poland (Bochnia, Bydgoszcz, Gdańskie, Kieleckie, Łódzkie, Lublin, Olsztyn, Poznań, Rzeszów, Rzeszowskie, Zielona Góra) [65,66,67]; Russia (North Caucasus, Smolensk) [70,75]; Serbia (Aleksa Šantić) [71]; South Korea (Jeju) [73]; USA (California, Florida, Georgia, Hawaii, Illinois, Iowa, Maryland, Minnesota) [25,26,75,80,104]; Zimbabwe (Harare) [85].

#### 3.1.13. *Eimeria suis* Nöller, 1921

Synonym: *Eimeria brumpti* Cauchemez, 1921 in part; *Eimeria debliecki* Douwes, 1921 in part [17]; *Eimeria jalina* (Perroncito, 1901) Neveu-Lemaire, 1912; *Eimeria perminuta* Henry, 1931 of Boch, Pezenburg and Rosenfeld (1961); *Eimeria tuis* Nöller, 1921 of Pellerdy (1963) [86].

Host: *Sus scrofa domestica* [26], *Sus scrofa scrofa* [97].

Founding location: Oocysts found in feces [17].

Geographic distribution: Australia (Queensland) [28]; Austria [97]; Brazil (Paraíba, Rio de Janeiro, Santa Catarina) [32,33,34]; China (Anhui, Beijing, Chongqing, Fujian, Guangdong, Guangxi, Guizhou, Hebei, Henan, Hunan, Jiangsu, Jiangxi, Qinghai, Shaanxi, Sichuan, Tianjin, Tibet, Yunnan, Zhejiang) [39,40,41,42,43,94,105]; Colombia (Caldas) [95]; Estonia [44]; France [45]; Germany (Hamburg, Lower Saxony) [46]; India (Karnātaka, Meghalaya, Punjab) [48,49,50]; Italy (Pisa) [51]; Japan (Ibaraki) [52]; Kenya (Western Province) [53]; Latvia [54]; Lithuania [55]; Nepal (Chitwan) [2]; Netherlands [57]; New Zealand (Wairarapa) [58]; Nigeria (Ibadan) [59]; Papua New Guinea (Port Moresby) [62]; Poland (North-west, Wielkopolska) [70,106]; Romania (Cluj) [13]; Russia (Smolensk) [70]; Tanzania (Morogoro) [76]; Ukraine [77]; United Kingdom (North Yorkshire) [78]; USA (Florida, Illinois, Kansas, Maryland, Oregon, Utah) [26,80,81,82,83]; Vietnam (Hanoi) [84]; Zimbabwe (Harare) [85].

#### 3.1.14. *Eimeria szechuanensis* Wu, Jiang and Hu, 1980

Host: *Sus scrofa domesticu* [18].

Founding location: Oocysts found in feces [18].

Geographic distribution: China (Chongqing, Jiangsu, Sichuan) [21].

#### 3.1.15. *Eimeria yanglingensis* Zhang, Yu, Feng and Li, 1994

Host: *Sus scrofa domesticus* [19].

Founding location: Oocysts found in feces [19].

Geographic distribution: China (Henan, Jiangxi, Shaanxi) [21,107]

#### 3.1.16. *Cystoisospora almataensis* Paichuk, 1953

Host: *Sus scrofa* [17], *Sus scrofa domesticus* [108].

Founding location: Oocysts found in feces [17].

Geographic distribution: China (Chongqing, Jiangsu, Inner Mongolia, Sichuan) [21]; Japan (Ibaraki) [52]; Kazakhstan (Alma-Ata) [26]; Moldova [51].

#### 3.1.17. *Cystoisospora suis* Biester, 1934

Synonym: *Isospora suis* Biester and Murray, 1934 [26]; *Isospora* sp. of Yakimoff, Iwanoff- Gobzem and Matschoulsky, 1936 [26]; *Cystoisospora suis* [32].

Host: *Sus scrofa* [97], *Sus scrofa domestica* [26], *Sus bucculentus* [27].

Parasitic site: Small intestine [26].

Geographic distribution: Argentina (Buenos Aires) [109]; Australia (New South Wales, Queensland, South Australia, Tasmania, Western Australia, Victoria) [110]; Austria [29]; Azerbaijan (Greater Caucasus) [30]; Bangladesh (Mymensingh) [111]; Belarus [27]; Belgium [4]; Brazil (Maranhão, Paraíba, Rio de Janeiro, Santa Catarina, Uberlândia) [31,32,33,34,35], Bulgaria [36]; Cameroon (Menoua) [112]; Canada (Ontario, Quebec) [37,113]; Chile [38], China (Anhui, Beijing, Chongqing, Fujian, Guangdong, Guangxi, Guizhou, Hebei, Henan, Heilongjiang, Hunan, Jiangsu, Jiangxi, Liaoning, Inner Mongolia, Shaanxi, Shandong, Shanxi, Sichuan, Tianjin, Tibet, Xinjiang, Yunnan, Zhejiang) [39,40,41,42,43,94,114,115,116,117]; Colombia (Caldas, Cundinamarca, Santander) [95,118,119]; Cuba (Villa Clara) [120]; Czech (Ceske Budejovice) [121]; Denmark [122]; Finland [123]; Germany (Hessen, Lower Saxony, Nordrhein-Westfalen, Westphalia) [46,124,125,126]; Ghana (Upper East Region) [127]; Greece [128]; Iceland [123]; India (Meghalaya) [49]; Italy (Pisa) [51]; Japan (Akita, Ibaraki, Iwate, Kumamoto, Nagasaki, Miyazaki) [12,52]; Kazakhstan (Alma-Ata) [21]; Latvia [54]; Lithuania [55]; Mexico (Chiapas) [129]; Moldova [56]; Myanmar (Nay Pyi Taw) [130]; Nepal (Chitwan) [2]; Netherlands [57]; New Zealand [131]; Nigeria (Abeokuta, Admawa, Ibadan) [59,60,132]; Norway [123]; Papua New Guinea (Port Moresby) [62]; Philippines (Batangas, Bulacan, Laguna, Quezon, Rizal) [133,134]; Poland (Bialystok, Bochnia, Gdańskie, Kieleckie, Łódzkie, Lubelskie, Rzeszowskie, Zielonogrskie) [64,65,66]; Russia (Udmurt) [23]; Serbia (Aleksa Šantić) [71]; South Korea (Jeju) [68]; Sweden [118]; Switzerland (North and South of the Alps) [135]; Uganda (Kamuli, Masaka, Mukono) [136]; Ukraine [77]; USA (Alabama, Florida, Georgia, Illinois, Iowa, Kansas, Maryland, South Dakota) [26,80,81,137,138,139,140]; Venezuela (Aragua, Carabobo, Yaracuy) [141,142,143].

#### 3.1.18. *Cystoisospora sundarbanensis* Ray and Sarkar, 1985

Host: *Sus scrofa* [20].

Founding location: Oocysts found in feces [20].

Geographic distribution: India (West Bengal) [20,90].

In this scoping review, 18 coccidia species from two genera found in the genus *Sus* were reported in a total of 63 countries, including 15 *Eimeria* and 3 *Cystoisospora*. Some previous reports showed that the *Cystoisospora* species infecting genus *Sus* was called *Isospora*. In the description of taxonomy, *Isospora* species oocysts had two sporocysts, each with four sporozoites and a single polar Stieda body, while *Cystoisospora* oocysts had two sporocysts, each with four sporozoites and no Stieda body in the sporocysts [144]. All *Isospora* species infecting mammals for which observations have been made possess oocysts that do not have a polar Stieda body in each sporocyst [144]. So, we used *Cystoisospora* instead of *Isospora* in this review; however, *Cystoisospora* and *Isospora* were used separately in the literature search.

The information about each species was introduced including its scientific name, synonym, host name, location, and geographical distribution. Synonyms refer to different scientific names given to the same taxonomic unit at different times. The identification of some coccidia species was not easy based on morphology before establishment of molecular detection methods; moreover, Coccidia species are almost always mixed infections, and it is difficult to isolate a single oocyst. So, some coccidia species have synonyms. In this review, the synonyms of some coccidia species were listed according to the book recorded by Levine (1988) and Donald and Lee Couch (1999) [17,86]. In fact, the location means the seat of the predilection of the parasite within the host. Many coccidia oocysts in feces were identified by morphology and molecular detection methods, without studying their life cycle in vivo, so the locations of these coccidia species within the host were not reported in the literature. Therefore, this review only describes oocysts found in feces.

Coccidia species in the genus *Sus* were different in different countries. In this review, 13 coccidia species were reported in China, 10 species in India, Japan, Lithuania, and Russia, 9 species in Brazil, Nigeria, Papua New Guinea, and USA, 8 species in Germany, Nepal, Ukraine, and Zimbabwe, 7 species in Australia and 6 other countries, 6 species in Chile and 6 other countries, 5 species in 4 countries, including Azerbaijan, 4 species in 5 countries including Bulgaria, 3 species in 3 countries including Czechia, 2 species in 9 countries including Belarus, and 1 species in 15 countries, including Argentina. Some countries have not reported or have reported fewer species of coccidia species in the genus *Sus*, possibly due to a lack of investigations and identification on coccidia.

Among the identified coccidia species in the genus *Sus*, the prevalence of different species is inconsistent in different countries. *C. suis* was found in 48 countries, *E. debliecki* in 45 countries, *E. scabra* in 33 countries, *E. polita* in 31 countries, *E. suis* in 28 countries, *E. perminuta* in 26 countries, *E. porci* in 24 countries, *E. neodebliecki* and *E. spinosa* in 21 countries each, *E. guevarai* in 5 countries, *C. almataensis* in 4 countries, *E. betica* in 2 countries, and *E. almataensis*, *E. ibrahimovae*, *E. residualis*, *E. szechuanensis*, *E. yanglingensis*, and *C. sundarbanensis* were each found in only 1 country. Based on the results, *C. suis*, *E. debliecki, E. scabra, E. polita, E. suis, E. perminuta*, *E. porci*, *E. neodebliecki,* and *E. spinosa* may be the main coccidia species in pigs. *C. suis* is regarded as the most prevalent gastrointestinal parasite in intensive pig farms [7]. Previous reports showed that *E. debliecki* was also the predominant species in China, Germany, Austria, and Switzerland [46,145]. However, the species composition in cases of porcine coccidia infection may vary depending on the region or the population studied.

In this review, some species synonyms were listed according to the references [17,86]. The appearance of synonyms may be due to the following reasons: (1) the original name was misspelled and later corrected by others; (2) with the improvement of microscope resolution, species previously named based on morphology were later found to be the same as a previously named species after further research, thus becoming a synonym of the identical species.

In addition, undefined coccidia species were reviewed in 20 countries (Table S2), including 11 countries with one or two species and 9 countries without coccidia species.

## 4. Conclusions

This review presents the detailed distribution of coccidia species in the genus *Sus* worldwide, thus providing a basis for understanding the species and geographical distribution of coccidia in the genus *Sus*. Limitations of the literature sources meant that the species and distribution of coccidia in the genus *Sus* in some countries were not included in this article; therefore, further studies are needed to supplement this information.

## Data Availability

The original contributions presented in this study are included in the article/Supplementary Materials. Further inquiries can be directed to the corresponding author.

## Supplementary Materials

The following supporting information can be downloaded at: https://www.mdpi.com/article/10.3390/microorganisms13010014/s1. Table S1: Extant species of genus Sus. Table S2: Countries reported unknown coccidia species in genus *Sus*. References [7,109,111,112,127,129,130,135,136,146,147,148,149,150,151,152,153,154,155,156,157,158,159,160,161,162,163,164] are cited in the supplementary materials.
