# Supplementary material for: Coccidia Species and Geographical Distribution in Genus Sus: A Scoping Review
Source: Microorganisms. 2024 Dec 25;13(1):14. doi: 10.3390/microorganisms13010014 (PMC11767909; doi:10.3390/microorganisms13010014)
Supplement: Supplementary file 1 [file microorganisms-13-00014-s001.zip › microorganisms-3345045-supplementary.pdf]

microorganisms

Table S1 Extant species of genus *Sus*

**Latin name**	**English name**
Sus ahoenobarbus Huet, 1888	Palawan bearded pig
Sus barbatus Müller, 1838	Bornean bearded pig
Sus cebifrons Heude, 1888	Visayan warty pig
Sus celebensis Müller & Schlegel, 1843	Celebes warty pig or Sulawesi warty pig
Sus oliveri Groves, 1997	Oliver's warty pig or Mindoro warty pig
Sus philippensis Nehring, 1886	Philippine warty pig
Sus scrofa or *Sus scrofa scrofa* Linnaeus, 1758	Wild boar
Sus scrofa domestica or *Sus domestica* Erxleben, 1777	Domestic pig
Sus verrucosus Boie, 1832	Javan warty pig

Table S2 Countries reported unknown coccidia species in genus *Sus*

Country and region	Infection rate of unknown coccidia species	Host	Reference
Argentina / Buenos Aires	Coccidia 40%	Domestic pigs / *Sus scrofa domestica*	Argañaraz *et al*（2018）[109]
	Eimeria sp. 18.5%, *Isospora* sp. 22%	Wild pigs / *Sus scrofa*	Ciocco *et al*（2019）[144]
Bangladesh	*Eimeria* spp. 56.4%	Domestic pigs / *Sus scrofa domestica*	Dey *et al*（2014）[111]
Cameroon s	Coccidia 26.9%	Domestic pigs / *Sus scrofa domestica*	Kouam *et al*（2018）[147]
	Eimeria spp. 1.4%	Domestic pigs / *Sus scrofa domestica*	Kouam and Ngueguim（2022）[112]
Croatia	*Eimeria* spp. 70.8%	Domestic pigs / *Sus scrofa domestica*	Salajpal *et al*（2017）[148]
Denmark	*Eimeria* spp. 92.3%	Wild pigs / *Sus scrofa*	Petersen *et al*（2020）[149]
Ethiopia	*Eimeria* spp. 4.89%~6.98%	Domestic pigs / *Sus scrofa domestica*	Abdu and Gashaw（2010）[150]
	Eimeria spp. 1.69%	Domestic pigs / *Sus scrofa domestica*	Tomass *et al*（2013）[151]
	Coccidia 11.98%	Domestic pigs / *Sus scrofa domestica*	Jufare *et al*（2015）[152]
Ghana	*Eimeria* spp. 77.2%	Domestic pigs / *Sus scrofa domestica*	Permin *et al*（1999）[127]
	Coccidia 14.5%	Domestic pigs / *Sus scrofa domestica*	Atawalna *et al*（2014）[153]
Guatemala	Coccidia 4.57%	Domestic pigs / *Sus scrofa domestica*	Cabrera Sarg（2017）[154]
Indonesia	*Eimiria* sp. 57.14%	Javan warty pigs / *Sus verrucosus*	Dewi and Nugraha（2007）[155]
	Eimeria sp. 78%	Domestic pigs / *Sus scrofa domestica*	Widisuputri *et al*（2020）[156]
Lesotho	Coccidia 27.78%	Domestic pigs / *Sus scrofa domestica*	Kompi *et al*（2023）[157]
Malaysia	Coccidia 11.6%	Domestic pigs / *Sus scrofa domestica*	Edmund *et al*（2005）[158]
Mexico	*Eimeria* sp. 52.63%	Wild pigs / *Sus scrofa*	Romero-Castañón *et al*（2008）[129]
Myanmar	*Eimeria* spp. 74%	Domestic pigs / *Sus scrofa domestica*	Bawm *et al*（2022）[130]
Portugal	*Eimeria* spp. 4.17%	Wild pigs / *Sus scrofa*	Figueiredo *et al*（2020）[159]
Rwanda	Coccidia 55.77%	Domestic pigs / *Sus scrofa domestica*	Tumusiime *et al*（2020）[160]
Slovakia	*Eimeria* spp. 90%	Wild pigs / *Sus scrofa*	Imrich *et al*（2016）[161]
	Eimeria spp. 8.33%	Wild pigs / *Sus scrofa*	Kanka *et al*（2017）[162]
Sweden	*Eimeria* spp. 9.23%	Domestic pigs / *Sus scrofa domestica*	Pettersson *et al*（2021）[7]
Switzerland	*Eimeria* sp. 74.5%	Wild pigs / *Sus scrofa*	Spieler and Schnyder（2021）[135]
Trinidad and Tobago	Coccidia 20.59%	Domestic pigs / *Sus scrofa domestica*	Adesiyun and Kaminjolo (1994) [163]
Uganda	*Eimeria* spp. 33.7%~50.8%	Domestic pigs / *Sus scrofa domestica*	Roesel *et al*（2017）[136]
	Eimeria spp. 56.4%	Domestic pigs / *Sus scrofa domestica*	Oba *et al*（2023）[164]
